# Neuroprotective and Anti-inflammatory Effects of Pioglitazone on Traumatic Brain Injury

**DOI:** 10.1155/2022/9860855

**Published:** 2022-06-17

**Authors:** Mohammad Yassin Zamanian, Niloofar Taheri, Maria Jade Catalan Opulencia, Dmitry Olegovich Bokov, Sharif Y. Abdullaev, Mohammadreza Gholamrezapour, Mahsa Heidari, Gholamreza Bazmandegan

**Affiliations:** ^1^Neurophysiology Research Center, Hamadan University of Medical Sciences, Hamadan 6718773654, Iran; ^2^Department of Pharmacology and Toxicology, School of Medicine, Hamadan University of Medical Sciences, Hamadan 6718773654, Iran; ^3^Student Research Committee, School of Medicine, Shahroud University of Medical Sciences, Shahroud, Iran; ^4^College of Business Administration Ajman University, Ajman, UAE; ^5^Institute of Pharmacy, Sechenov First Moscow State Medical University, 8 Trubetskaya St. bldg. 2, Moscow 119991, Russia; ^6^Laboratory of Food Chemistry, Federal Research Center of Nutrition, Biotechnology and Food Safety, 2/14 Ustyinsky pr, Moscow 109240, Russia; ^7^Department of Maxillo-facial diseases and Traumatology, Tashkent State Dental Institute, Makhtumkuli, 103 Tashkent, Uzbekistan; ^8^Clinical Research Development Unit, Ali-Ibn Abi-Talib Hospital, Rafsanjan University of Medical Sciences, Rafsanjan, Iran; ^9^Department of Internal Medicine, Ali-Ibn Abi-Talib Hospital, School of Medicine, Rafsanjan University of Medical Sciences, Rafsanjan, Iran; ^10^Department of Biochemistry, Institute of Biochemistry and Biophysics (IBB), University of Tehran, Tehran, Iran; ^11^Department of Family Medicine, Ali-Ibn Abi-Talib Hospital, School of Medicine, Rafsanjan University of Medical Sciences, Rafsanjan, Iran

## Abstract

Traumatic brain injury (TBI) is still a major cause of concern for public health, and out of all the trauma-related injuries, it makes the highest contribution to death and disability worldwide. Patients of TBI continue to suffer from brain injury through an intricate flow of primary and secondary injury events. However, when treatment is provided in a timely manner, there is a significant window of opportunity to avoid a few of the serious effects. Pioglitazone (PG), which has a neuroprotective impact and can decrease inflammation after TBI, activates peroxisome proliferator-activated receptor-gamma (PPAR*γ*). The objective of the study is to examine the existing literature to assess the neuroprotective and anti-inflammatory impact of PG in TBI. It also discusses the part played by microglia and cytokines in TBI. According to the findings of this study, PG has the ability to enhance neurobehavior, decrease brain edema and neuronal injury following TBI. To achieve the protective impact of PG the following was required: (1) stimulating PPAR*γ*; (2) decreasing oxidative stress; (3) decreasing nuclear factor kappa B (NF-*κ*B), interleukin 6 (IL-6), interleukin-1*β* (IL-1*β*), cyclooxygenase-2 (COX-2), and C-C motif chemokine ligand 20 (CCL20) expression; (4) limiting the increase in the number of activated microglia; and (5) reducing mitochondrial dysfunction. The findings indicate that when PIG is used clinically, it may serve as a neuroprotective anti-inflammatory approach in TBI.

## 1. Introduction

Traumatic brain injury (TBI) is responsible for numerous deaths and permanent disability associated with the advancement of secondary brain injury and bleeding, due to which there is an increase in the rate of morbidity and mortality [1]. Every year, around 3.17 million cases of deaths and disabilities related to TBI are experienced [2].

For a long time, TBI has been considered as a “silent epidemic” in society as it is one of the main causes of death and disability among individuals of young age in Western nations [3].There has been a rapid increase in the incidence of TBI because of a significant increase in the number of road accidents, e.g., motor vehicle accidents [4, 5].

It is possible to categorize TBI as mild, moderate, or severe. Almost 80 to 90% of all TBIs are mild in nature. There is a low percentage of severe TBI; however, it involves a high rate of mortality of between 30 and 40% [6, 7].

TBI consists of complex mechanisms and a cascade network, ranging from physical primary cerebral trauma to various secondary injuries [8].

The physiopathological processes of TBI need to be additionally explained, and the latest efficient pharmacological targets for treating TBI need to be determined. The pathophysiology of TBI is known to involve various kinds of pathological and physiological modifications, which typically comprise of primary and secondary brain injury that cause neurological deficits, neuronal death, and mortality following TBI [9].

It has been found that brain edema, vascular dysfunction, blood-brain barrier (BBB) disruption, and glial responses are related to the occurrence of secondary injuries after encountering TBI, which eventually gives rise to irreversible neuronal injury or possibly death [10].

It is vital to note that different kinds of cells, including endothelial cells, neurons, and astrocytes, are involved in the pathological processes of TBI [11].

It is quite challenging to prevent primary brain injury, which refers to a physical injury experienced directly by the brain tissue, and if encountered, it cannot typically be reversed, leading to brain tissue disorganization, intracerebral hemorrhage, and damage to BBB.

Secondary brain injury involves oxidative stress, calcium overload, autophagy, neuroinflammation, lipid peroxidation, necroptosis, and apoptosis and is possible to reverse [12, 13]. There are complicated mechanisms involved in secondary brain injury, which are characterized by changes in cerebral perfusion, triggering of inflammatory cytokines, and excitotoxicity [14].

So far, the focus of preclinical and clinical studies has mainly been on secondary injury processes so that the progression of injury pathology can be slowed down, thus decreasing cellular stress on glia and neurons [15].

Microglia and astrocytes are activated after encountering TBI, which leads to the overproduction of neuroinflammatory mediators that intensify TBI. Hence, the therapeutic targets for treating TBI could be determined by identifying the particular mechanisms of TBI [16, 17]. Following TBI, neuroinflammation plays a vital part in secondary tissue damage, which results in neuronal damage and dysfunction. Currently, the most widely used treatment method is surgical intervention, but there is no successful treatment for secondary brain injury following TBI. Hence, it is imperative to formulate new and effective methods through which neuroinflammation following TBI could be treated and the prognosis of patients could be enhanced [18, 19].

A lot of research has been carried out on formulating therapeutic strategies against TBI-induced neurodegeneration; however, it is still a significant public health issue in all age groups all over the world, irrespective of the financial status and income level of the patient [20].

The focus of previous studies has been on targeting proliferator-activated receptor-*γ* (PPAR-*γ*) to regulate neuroinflammation by reducing the generation of proinflammatory mediators in microglia and macrophages due to neurological injury [21].

Thiazolidinediones, or glitazones, are a group of drugs that have been studied extensively in the area of neuronal insult and injury [22].

The capability of PG, a selective agonist of the PPAR*γ*, to treat neuronal injury and neuroinflammation following diffuse brain injury, was also examined by the researchers [19]. PG signifies an insulin-sensitizing drug that has been approved for treating T2D. The PPAR*γ*, a transcriptional regulator of adipocyte differentiation and lipid storage which is found in abundance in adipose tissue, mainly regulates the molecular impact of PG [23].

It has been shown by researchers that anti-inflammatory and neuroprotective properties are exhibited by PG as PPAR*γ* receptor activation [24–26].

The preliminary reports on the use of PG and other “glitazones” as a therapeutic agent for TBI from preclinical TBI studies concentrate on possible anti-inflammation effects regulated through PPAR that could suppress secondary injury cascades. Rosiglitazone and other PPAR agonists, like fenofibrate, have been found to alleviate inflammation and oxidative damage following experimental TBI to offer neuroprotection [15, 27, 28]. Furthermore, PG decreases a secondary inflammatory response in the ischemic insult to some extent, particularly in the endothelial and perivascular tissues of rats [29].

PPAR*γ* agonists like PG not only have an impact on metabolic disorders but also regulate inflammatory responses, such as immune function in the central nervous system [30].

The neuroprotective and anti-inflammatory impact of PG on TBI was examined in this study. Therefore, the latest studies carried out on the part played by PG in treating and managing TBI were identified, and the underlying approaches followed were discussed. The neuroprotective and anti-inflammatory effects were specifically examined.

## 2. Types of TBI

Various systems can be used to examine the severity of TBI, and the most extensively used system in this regard is the Glasgow Coma Scale (GCS). TBI is categorized by the GCS into mild (GCS range of 13 to 15), moderate (GCS in the range of 9 to 12), and severe (GCS in the range of 3 to 8), based on the scores gained from particular clinical assessments carried out in other contexts, for example verbal communication, eye-opening, and motor functions [31].

There are three groups into which TBI can be classified, depending on its unique physical insult: closed head, penetrating, and explosive blast TBI. The closed-head TBI is the most widely prevalent incident among civilians that is caused due to blunt objects, falling, sports injuries, and vehicle accidents. Penetrating TBI refers to a brain injury in which a foreign body enters the brain parenchyma, leading to focal damage, intracranial hemorrhage, ischemic conditions, and edema [32, 33].

A type of TBI that occurs due to an explosion is known as explosive TBI. This type of TBI is a war-related TBI that was recognized initially in the 20th century [34]. The brain is seriously affected in an explosion blast TBI due to rapid pressure shock waves, and a substantial amount of energy is transferred from the skull to the brain parenchyma [34].

## 3. Pathophysiology of TBI

There are two groups into which the pathophysiological outcomes of TBI can be distinguished: primary brain injury and secondary brain injury [35].

## 4. Primary Brain Injury

Primary brain injury is experienced at the point the trauma occurs. Some of the common mechanisms involve rapid acceleration/deceleration, direct impact, penetrating injury, and blast waves. Those chronic inflammatory procedures that extend for a few months or years following primary brain injury may lead to neurodegeneration, cell death, and neurological disabilities [35].

There is physical damage to intracranial structures in primary brain injury. In this injury, direct damage is caused to brain parenchyma, like hematomas, contusions, diffuse axonal injury, and lacerations. In addition, direct vascular damage is also experienced, which causes hemorrhage and vasogenic edema [36].

The most serious kind of primary brain injury is laceration. It is likely to experience axial hematomas in the brain parenchyma and extra-axial hematomas within the subarachnoid, subdural, and epidural spaces, due to which brain compression and serious neurological damage may occur [36].

## 5. Secondary Brain Injury

It is suggested by alterations in the neurovascular unit that secondary injuries have occurred in the brain following TBI. Secondary brain injury consists of neuroimmune and inflammatory responses that may be experienced within days, weeks, months, or several years following the initial brain damage after TBI [37, 38].

**Figure 1:**
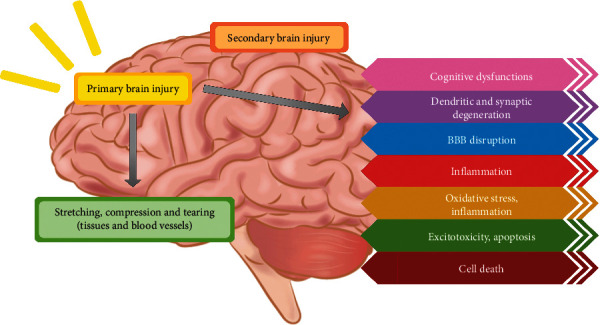
Effects of TBI types on the brain.

Secondary intracranial injury is mainly regulated by increased activity of excitatory neurotransmitters, production of reactive oxygen species (ROS), mitochondria dysfunction, and creation of proinflammatory cytokines, and all of these can play a part in neuronal cell damage and potentially lead to cell death. After secondary damage to neuronal tissue, cerebral edema production, greater intracranial pressure (ICP), impairment of the blood-brain barrier (BBB), and changes in cerebrovascular reactivity may occur [36, 39]. The harmful effects of various types of TBI are shown in Figure 1.

## 6. Traumatic Brain Injury and Neuroinflammation

Inflammation refers to a local response made by mammalian tissue to an injury brought about by any agent and involves pain, redness, swelling, and heat production [40–42].

The permeability of the blood-brain-barrier (BBB) increases the infiltration of circulating monocytes, lymphocytes, and neutrophils inside the brain parenchyma within 24 hours of TBI. Because of this, the complement system is stimulated and the inflammatory cytokines like tissue necrosis factor-*α* (TNF-*α*), interleukin-6 (IL-6), and interleukin-1*β* (IL-1*β*) that are proinflammatory cytokines are released [43–46]. When these inflammatory cytokines are released, BBB dysfunction and brain edema occur. It was determined that following injury, the stimulated microglia and macrophages get to the site of injury to decrease the damaging effects of the brain injury and offer a secure environment [47].

The particular resident macrophage of the central nervous system (CNS) that has phagocytosis and antigen presentation abilities is known as microglia. Microglia constitute the most widely prevalent mononuclear phagocyte inside the CNS and are responsible for around 10% of the overall CNS cell population in adults [48].

The release of inflammatory cytokines, reactive oxygen species, and glutamate may be brought about by the activated microglia, which increases the severity of the injury and causes neuronal cell death [31, 49].

The current grouping of myeloid cells (such as macrophages, monocytes, and microglia) into M1 and M2 polarization states occurs in accordance with the culturing of these cells in vitro, which stimulates them with individual cytokines, e.g., IFN-*γ*, IL-4, IL-10, or IL-13 [50].

The two phenotypes of activated microglial cells play a significantly different role: the M1 phenotype is responsible for the release of numerous proinflammatory cytokines, like TNF-*α*, and the M2 phenotype is found to be associated with the release of anti-inflammatory cytokines, such as IL-10, and be involved in neural regeneration processes, e.g., neurogenesis, angiogenesis, oligodendrogenesis, and remyelination [51].

Pharmacological approaches that inhibit the M1 phenotype and release the M2 phenotype of microglial cells in animal models may help in decreasing cerebral damage and improving neurological function recovery after TBI [52].

Due to the action of proinflammatory molecules of microglia, they are directed towards the inflammatory M1 phenotype that creates the proinflammatory cytokines (TNF*α*, IL-1*β*, IL-6, -12, -18, -23, and IFN*γ*) and chemokines (CCL2, 5 and 20, CXCL1, and 9 and 10), and also reactive oxygen species (ROS). On the other hand, microglia follow an alternative activation phenotype that is referred to as M2 in reaction to anti-inflammatory cytokines (IL-4, -10 and -13, and TGF*β*). There are anti-inflammatory properties of this phenotype, which is involved in tissue restoration and regeneration [51, 53].

## 7. Neuroprotective and Anti-inflammatory Effects of Pioglitazone

In secondary tissue damage, a major part is played by neuroinflammation following TBI, which gives rise to neuronal damage and impairment [19].

Anti-inflammatory and antioxidant effects are shown by PPAR agonists in various types of CNS disorders, for example Alzheimer's, ischemic stroke, and Parkinson's disease [54–56]. Transrepression of the redox-mediated transcription factor nuclear factor kappa B (NF-*κ*B) is used to regulate the protective anti-inflammatory impact of PPAR*γ* to some extent [57, 58].

Various molecules take part in the initial stages of the immune response, and NF-*κ*B mediates different stages of the inflammatory response, which include IL-1*β*, IL-6, adhesion molecules, chemokines, and colony-stimulating factors [59, 60].

Interleukin-6 (IL-6) takes part in different physiological activities, such as hematopoiesis, immunity, neurodevelopment, and bone metabolism [61].

It was shown by Deng et al. that PG was able to effectively decrease neuroinflammation following TBI and the intensity of cerebral edema, as well as support neurological recovery following inflammation. To some extent, the beneficial impact of PG may be dependent on PPAR*γ* activation, PPAR*γ*/NF-*κ*B/IL-6 signaling pathway that performs a vital regulatory role in treating TBI with PG. According to this data, PG has the potential to be used as a therapeutic method of treating TBI [19].

Neuroprotective effects have been shown by PG, which is one of the glitazones, by decreasing mitochondrial dysfunction [62].

It was hypothesized in earlier studies that neuroinflammation modulation, depending on interactions with PPAR-*γ*, regulated the therapeutic effect of PG [62, 63].

It was demonstrated by Yonutas et al. that mitochondrial dysfunction can be reinstated by PG (10 mg/kg) when it is administered at 12 hours following a TBI. This study showed that PG can bring increments in mitochondrial bioenergetics by more than 54% in wild-type mice that express mitoNEET in their mitochondria, in comparison to vehicle-treated. The study findings also show the vital role of mitoNEET (which is an iron-containing external mitochondrial membrane protein that controls oxidative potential) for mitochondrial bioenergetics, as well as its significance in the neuropathological sequelae of TBI. In addition, the study shows the importance of mitoNEET for PG-regulated neuroprotection [22].

Inhibitory postsynaptic potentials are created by dopamine, which is also very important in motivation, learning, and movement [64]. According to Liu et al., dopaminergic neurodegeneration and inflammation was triggered in the substantia nigra, identical to certain pathological characteristics of Parkinson's disease, by a single moderate brain injury caused by midline fluid percussion brain injury (mFPI).

It was determined that PG (10 mg/kg) supported dopaminergic neuronal survival and locomotor functional recovery, and this may be related to the decrease in microglial activation. This study showed that the inflammatory reaction determined in the nigrostriatal system had a role in the secondary pathology of TBI.

It was found by the researchers that a short while following brain injury (within 6 hours), there was significant production of proinflammatory cytokines, e.g., TNF*α*, IL-1*β*, IL-6, and CXCL1 in the striatum and substantia nigra. Cytokines represent small, short-lived proteins that are created by blood leukocytes and glial cells. To sum up, treatment with PG significantly weakened microglial activation and enhanced dopaminergic neuronal survival in the nigrostriatal system, which may support locomotor recovery. It is indicated by these findings that a suitable therapeutic treatment to enhance the outcome following TBI may be interventions that decrease secondary inflammation [3].

Both in vitro and in vivo models have been used in various studies to show PPAR-regulated decrease in the release of proinflammatory cytokines and oxidative stress markers [65–68].

Perilesional edema corresponded with inflammation in preclinical studies and involved the generation of proinflammatory cytokines, migration of peripheral immune cells to the brain, and activation of resident brain astrocytes [69].

From the various inflammasomes in the brain, the cleavage of caspase-1 and interleukin-1*β* (IL-1*β*) is supported by nucleotide-binding domain and leucine-rich repeat (NLR) family pyrin domain-including protein 3 (NLP3), which enhances the inflammatory response [70, 71].

It has been determined from the latest studies on humans and rodents that after TBI, NLRP3-related molecules are upregulated [72].

Yi et al. found in their study that a vital part is played by NLRP3 inflammasome in TBI, particularly in cerebral edema and secondary inflammation. Furthermore, cerebral edema and inflammation were decreased by PG (10, 20, and 40 mg/kg) through the downregulation of NLRP3-related inflammasomes. Hence, NLRP3 is a potential therapeutic target for TBI, and a valuable part may be played by the clinical application of PG in treating TBI by downregulating NLRP3 and restricting astrocyte and microglial activity [73].

It has also been found that in various tissues, PG decreases proinflammatory cytokines like IL1-*β* and C-C motif chemokine ligand 20 (CCL20) [74, 75]. Earlier studies also reported that the proinflammatory chemokine CCL20 is created in the degenerating cerebral tissues following TBI [76, 77].

BDNF is a neurotrophic factor that is related to post-TBI depression and cognitive dysfunction [78, 79].

According to Das et al., TBI in rats brought about microglial and astroglial activation, enhanced secretion of proinflammatory cytokines like CCL20 and IL1-*β*, and led to behavioral and sensorimotor deficiencies. Neuroinflammation in the brain was decreased by PG (2 mg/kg in 100 *μ*L) by reducing inflammatory cytokine production before the transplantation of human mesenchymal stem cells (hMSCs). The efficiency of the transplanted hMSC was enhanced by the decrease in local cerebral inflammation, which was clear from the greater neurogenesis, decreased anxiety-like behavior, and lower pain sensation in rats undergoing combination treatment. In a reduced inflammatory microenvironment, hMSCs potentially assist in histological and behavioral recovery by increasing the production of neurotropic factors such as BDNF [76].

TBI was followed by a major disruption of mitochondrial homeostasis, which led to a decrease in cellular bioenergetics, increase in mitochondrial ROS production, and decrease in synaptic equilibrium. Hence, after TBI, a significant factor that determines cell survival or death may be the extent of mitochondrial injury or dysfunction [62, 80].

PPAR*γ* agonist PG also reduces oxidative damage, mitochondrial dysfunction, and cell death [81, 82].

It was demonstrated by Sauerbeck et al. that PG treatment (10 mg/kg) did not allow the number of activated microglia in rats to increase. The authors deduced from these studies that after TBI, PG has the ability to improve various areas of neuropathology. The experiments demonstrate that PG can protect mitochondria, decrease inflammation, and enhance cognitive function after encountering TBI. Additional support is offered in these studies for offering neuroprotection by using PPAR ligands, particularly PG [62].

According to Qiu et al., twenty-four hours following TBI, there was a significant upregulation in the expression of PPAR*γ* mRNA in all PG groups, with a substantial disparity among every PG group, depending on the dose concentration. There was substantial downregulation of the expression of TNF-*α* mRNA in the treatment groups following injury. Twenty-four hours following the injury, the levels of TNF-*α* and IL-6 mRNAs in the PG groups getting doses of 1.0 and 10.0 mg/kg decreased in comparison to the groups that were given 0.5 mg/kg PG. To sum up, a decrease in the levels of inflammatory cytokines in rats with TBI is caused by PG through the upregulation of PPAR*γ* [83].

An inducible isoform of COX, COX-2 transforms arachidonic acid into precursors of distinct prostaglandins that play a significant part in regulating cerebral circulation and neuronal signaling [84].

Lipid peroxidation refers to the oxidative degradation of polyunsaturated fatty acids that caused damage to the cellular membrane phospholipids and eventually led to cell dysfunction in various mammalian tissues, such as the brain [85].

It was determined by Pilipović et al. that PG (1 mg/kg) at 10 min following TBI brought about a substantial decline in the cortical lipid and protein oxidative damage, raised the GSH-Px activity, and decreased microglial response. The tested PPAR*γ* agonist did not cause any change in the cortical reactive astrocytosis in injured animals (rats). It is shown by these results that PG that is given in a single dose, early on after lateral fluid percussion injury (LFPI), brought about a decrease in cortical oxidative damage, improved antioxidant defense, and showed little anti-inflammatory impact [86].

Thal et al. showed in their study that in a mouse model of TBI, secondary brain damage is decreased by PG (1 mg/kg). Hence, PPAR-c-independent anti-inflammatory functions in the brain offer neuroprotection to a certain degree. The main outcomes of the study were as follows: (1) when given 30 minutes following brain injury, PG decreased brain contusion volume; (2) cerebral inflammation was prevented by PG by decreasing the expression of TNF-*α*, IL1-*β*, and IL-6 gene in brain tissue; and (3) PPAR-c suppression taking place simultaneously did not revoke the protective impact of PG [63].

## 8. Conclusion

**Figure 2:**
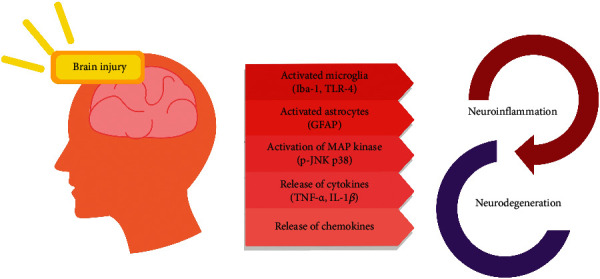
Neuroprotective and anti-inflammatory effects of the PG.

The increasing evidence regarding the neuroprotective and the anti-inflammatory impact of PG on TBI is discussed in this review. Though these studies were carried out in animal models of TBI, it was possible to get similar findings in human TBI patients (Table 1). It is demonstrated in the study that PG is highly capable of decreasing neurodegeneration and enhancing the functional consequences in TBI patients by decreasing inflammation. PG has the therapeutic potential to decrease inflammation, reduce oxidative stress, reduce lesion volume, and enhance behavioral outcome after encountering TBI. On the basis of the findings of this study, the PPAR*γ*/NF-*κ*B/IL-6 pathway mainly regulated the neuroprotective impact of PG on TBI. Nevertheless, it is important to carry out additional studies to determine the clinical impact of PG on TBI and their molecular methods.  Figure 2 presents a summary of the neuroprotective and anti-inflammatory effect of the PG.

## Figures and Tables

**Table 1:** Studies consistent with the purpose of this study.

Authors	Dosage of PG	Effects
Deng et al.	1.0 mg/kg	Reduces neuroinflammation, reduces the extent of cerebral edema, and promotes neurological recovery
Yonutas et al.	10 mg/kg	Restores mitochondrial dysfunction and increase mitochondrial bioenergetics
Liu et al.	10 mg/kg	Promotes dopaminergic neuronal survival and locomotor functional recovery
Yi et al.	10, 20, and 40 mg/kg	Reduces cerebral edema and inflammation by downregulating NLRP3-related inflammasomes
Das et al.	2 mg/kg	Reduces neuroinflammation in the brain by decreasing inflammatory cytokine production prior to hMSC transplantation
Sauerbeck et al.	10 mg/kg	Protects mitochondria, reduces inflammation, minimizes the cortical lesion, and improves cognitive function
Qiu et al.	1.0 and 10.0 mg/kg	Decreases the levels of inflammatory cytokines via upregulating PPAR*γ*
Pilipović et al.	1 mg/kg	Reduces cortical oxidative damage, increased antioxidant defense, and had limited anti-inflammatory effect
Thal et al.	1 mg/kg	Reduces brain contusion volume; suppressed cerebral inflammation by reducing TNF-*α*, IL1-*β*, and IL-6 gene expression in brain tissue and simultaneous PPAR-c inhibition

## Abbreviations

BBB: Blood-brain barrier
BDNF: Brain-derived neurotrophic factor
CCL2: C-C motif chemokine ligand 2
CCL20: C-C motif chemokine ligand 20
CNS: Central nervous system
COX-2: Cyclooxygenase-2
CXCL1: C-X-C motif chemokine ligand 1
CXCL9: C-X-C motif chemokine ligand 9
CXCL10: C-X-C motif chemokine ligand 10
GCS: Glasgow Coma Scale
GSH-Px: Plasma glutathione peroxidase
hMSCs: Human mesenchymal stem cells
ICP: Intracranial pressure
IFN*γ*: Interferon gamma
IL-1*β*: Interleukin-1beta
IL-4: Interleukin 4
IL-6: Interleukin 6
IL-10: Interleukin 10
IL-12: Interleukin 12
IL-13: Interleukin 13
IL-18: Interleukin 18
LFPI: Lateral fluid percussion injury
mFPI: Midline fluid percussion brain injury
NF-*κ*B: Nuclear factor kappa B
NLRP3: Nucleotide-binding domain leucine-rich repeat (NLR) family pyrin domain–containing protein 3
PG: Pioglitazone
PPAR-*γ*: Peroxisome proliferator-activated receptor-gamma
ROS: Reactive oxygen species
TBI: Traumatic brain injury
TGF*β*: Transforming growth factor-beta
TNF-*α*: Tissue necrosis factor-alpha.

## References

[1] Li X.-L., Wang B., Yang F.-B., Chen L.-G., You J. (2022). HOXA11-AS aggravates microglia-induced neuroinflammation after traumatic brain injury. *Neural Regeneration Research*.

[2] Zamanian M. Y., Kujawska M., Nikbakhtzadeh M. (2021). Carvacrol as a potential neuroprotective agent for neurological diseases: a systematic review article. *CNS & Neurological Disorders Drug Targets*.

[3] Liu M., Bachstetter A. D., Cass W. A., Lifshitz J., Bing G. (2017). Pioglitazone attenuates neuroinflammation and promotes dopaminergic neuronal survival in the nigrostriatal system of rats after diffuse brain injury. *Journal of Neurotrauma*.

[4] Dunne J., Quiñones-Ossa G. A., Still E. G. (2020). The epidemiology of traumatic brain injury due to traffic accidents in Latin America: a narrative review. *Journal of neurosciences in rural practice*.

[5] Hu Y., Feng X., Chen J., Wu Y., Shen L. (2022). Hydrogen-rich saline alleviates early brain injury through inhibition of necroptosis and neuroinflammation via the ROS/HO-1 signaling pathway after traumatic brain injury. *Experimental and Therapeutic Medicine*.

[6] Blennow K., Brody D. L., Kochanek P. M. (2016). Traumatic brain injuries. *Nature Reviews. Disease Primers*.

[7] Maas A. I. R., Menon D. K., Adelson P. D. (2017). Traumatic brain injury: integrated approaches to improve prevention, clinical care, and research. *Lancet Neurology*.

[8] Bramlett H. M., Dietrich W. D. (2015). Long-term consequences of traumatic brain injury: current status of potential mechanisms of injury and neurological outcomes. *Journal of Neurotrauma*.

[9] Wang Y., Wang L., Hu T. (2020). Hydrogen improves cell viability partly through inhibition of autophagy and activation of PI3K/Akt/GSK3*β* signal pathway in a microvascular endothelial cell model of traumatic brain injury. *Neurological Research*.

[10] Lu Q., Xiong J., Yuan Y. (2022). Minocycline improves the functional recovery after traumatic brain injury via inhibition of aquaporin-4. *International Journal of Biological Sciences*.

[11] Watkins S., Robel S., Kimbrough I. F., Robert S. M., Ellis-Davies G., Sontheimer H. (2014). Disruption of astrocyte-vascular coupling and the blood-brain barrier by invading glioma cells. *Nature Communications*.

[12] Li H., Lu C., Yao W., Xu L., Zhou J., Zheng B. (2020). Dexmedetomidine inhibits inflammatory response and autophagy through the circLrp1b/miR-27a-3p/Dram2 pathway in a rat model of traumatic brain injury. *Aging (Albany NY)*.

[13] Wang Y., Zhao M., Shang L. (2020). Homer1a protects against neuronal injury via PI3K/AKT/mTOR signaling pathway. *The International Journal of Neuroscience*.

[14] Cornelius C., Crupi R., Calabrese V. (2013). Traumatic brain injury: oxidative stress and neuroprotection. *Antioxidants & Redox Signaling*.

[15] Hubbard W. B., Spry M. L., Gooch J. L. (2021). Clinically relevant mitochondrial-targeted therapy improves chronic outcomes after traumatic brain injury. *Brain*.

[16] Kabadi S. V., Stoica B. A., Loane D. J., Luo T., Faden A. I. (2014). CR8, a novel inhibitor of CDK, limits microglial activation, astrocytosis, neuronal loss, and neurologic dysfunction after experimental traumatic brain injury. *Journal of Cerebral Blood Flow and Metabolism*.

[17] Sharma R., Kambhampati S. P., Zhang Z. (2020). Dendrimer mediated targeted delivery of sinomenine for the treatment of acute neuroinflammation in traumatic brain injury. *Journal of Controlled Release*.

[18] Hegdekar N., Lipinski M. M., Sarkar C. (2021). _N_ -Acetyl-l-leucine improves functional recovery and attenuates cortical cell death and neuroinflammation after traumatic brain injury in mice. *Scientific Reports*.

[19] Deng Y., Jiang X., Deng X. (2020). Pioglitazone ameliorates neuronal damage after traumatic brain injury via the PPAR*γ*/NF-*κ*B/IL-6 signaling pathway. *Genes & diseases*.

[20] Ng S. Y., Lee A. Y. W. (2019). Traumatic brain injuries: pathophysiology and potential therapeutic targets. *Frontiers in Cellular Neuroscience*.

[21] Kiaei M. (2008). Peroxisome proliferator-activated receptor-gamma in amyotrophic lateral sclerosis and Huntington's disease. *PPAR Research*.

[22] Yonutas H. M., Hubbard W. B., Pandya J. D., Vekaria H. J., Geldenhuys W. J., Sullivan P. G. (2020). Bioenergetic restoration and neuroprotection after therapeutic targeting of mitoNEET: new mechanism of pioglitazone following traumatic brain injury. *Experimental Neurology*.

[23] Palavicini J. P., Chavez-Velazquez A., Fourcaudot M. (2021). The insulin-sensitizer pioglitazone remodels adipose tissue phospholipids in humans. *Frontiers in Physiology*.

[24] Zhao Y., Lützen U., Gohlke P., Jiang P., Herdegen T., Culman J. (2021). Neuroprotective and antioxidative effects of pioglitazone in brain tissue adjacent to the ischemic core are mediated by PI3K/Akt and Nrf2/ARE pathways. *Journal of Molecular Medicine (Berlin, Germany)*.

[25] El-Sahar A. E., Safar M. M., Zaki H. F., Attia A. S., Ain-Shoka A. A. (2015). Neuroprotective effects of pioglitazone against transient cerebral ischemic reperfusion injury in diabetic rats: modulation of antioxidant, anti- inflammatory, and anti-apoptotic biomarkers. *Pharmacological Reports*.

[26] Swanson C. R., Joers V., Bondarenko V. (2011). The PPAR-*γ* agonist pioglitazone modulates inflammation and induces neuroprotection in parkinsonian monkeys. *Journal of Neuroinflammation*.

[27] Yi J. H., Park S. W., Brooks N., Lang B. T., Vemuganti R. (2008). PPAR*γ* agonist rosiglitazone is neuroprotective after traumatic brain injury via anti-inflammatory and anti-oxidative mechanisms. *Brain Research*.

[28] Hyong A., Jadhav V., Lee S. (2008). Rosiglitazone, a PPAR gamma agonist, attenuates inflammation after surgical brain injury in rodents. *Brain Research*.

[29] Pane B., Gazzola V., Spinella G. (2018). Inflammatory response modulation through a PPAR*γ* agonist during surgically induced visceral ischemia in an animal model. *Annals of Vascular Surgery*.

[30] Kapadia R., Yi J. H., Vemuganti R. (2008). Mechanisms of anti-inflammatory and neuroprotective actions of PPAR-gamma agonists. *Frontiers in Bioscience*.

[31] Ikram M., Park H. Y., Ali T., Kim M. O. (2021). Melatonin as a potential regulator of oxidative stress, and neuroinflammation: mechanisms and implications for the management of brain injury-induced neurodegeneration. *Journal of Inflammation Research*.

[32] Magnuson J., Leonessa F., Ling G. S. (2012). Neuropathology of explosive blast traumatic brain injury. *Current Neurology and Neuroscience Reports*.

[33] Ling G., Ecklund J. M., Bandak F. A. (2015). Brain injury from explosive blast: description and clinical management. *Handbook of Clinical Neurology*.

[34] Ling G. S., Ecklund J. M. (2011). Traumatic brain injury in modern war. *Current Opinion in Anesthesiology*.

[35] Marjani S., Zirh S., Sever-Bahcekapili M., Cakir-Aktas C., Muftuoglu S. F., Mut M. (2021). Doxycycline alleviates acute traumatic brain injury by suppressing neuroinflammation and apoptosis in a mouse model. *Journal of Neuroimmunology*.

[36] Sande A., West C. (2010). Traumatic brain injury: a review of pathophysiology and management. *Journal of Veterinary Emergency and Critical Care*.

[37] Crupi R., Cordaro M., Cuzzocrea S., Impellizzeri D. (2020). Management of traumatic brain injury: from present to future. *Antioxidants (Basel)*.

[38] Kempuraj D., Ahmed M. E., Selvakumar G. P. (2021). Acute traumatic brain injury-induced neuroinflammatory response and neurovascular disorders in the brain. *Neurotoxicity Research*.

[39] Sahel D. K., Kaira M., Raj K., Sharma S., Singh S. (2019). Mitochondrial dysfunctioning and neuroinflammation: recent highlights on the possible mechanisms involved in traumatic brain injury. *Neuroscience Letters*.

[40] Banerjee P., Kumar T., Sarangi S. C., Meetei U. D., Devi A. S., Kumar R. (2021). Anti-inflammatory potential of aqueous extract of Elsoltzia stachyodes on experimental models of inflammation in rats. *Journal of Natural Science, Biology and Medicine*.

[41] You S.-H., Yoon M.-Y., Moon J.-S. (2021). Antioxidant and anti-inflammatory activity study of fulvic acid. *Journal of Natural Science, Biology and Medicine*.

[42] Subramanian A. K., Prabhakar R., Vikram N. R., Dinesh S. S., Rajeshkumar S. (2021). In vitro anti-inflammatory activity of silymarin/hydroxyapatite/chitosan nanocomposites and its cytotoxic effect using brine shrimp lethality assay. *Journal of Population Therapeutics and Clinical Pharmacology*.

[43] Lotocki G., de Rivero Vaccari J. P., Perez E. R. (2009). Alterations in blood-brain barrier permeability to large and small molecules and leukocyte accumulation after traumatic brain injury: effects of post-traumatic hypothermia. *Journal of Neurotrauma*.

[44] Zhuang S., Liu B., Guo S. (2021). Germacrone alleviates neurological deficits following traumatic brain injury by modulating neuroinflammation and oxidative stress. *BMC Complementary Medicine and Therapies*.

[45] Rahmani M.-R., Shamsizadeh A., Moghadam-Ahmadi A., Bazmandegan G., Allahtavakoli M. (2018). JZL184, as a monoacylglycerol lipase inhibitor, down-regulates inflammation in a cannabinoid pathway dependent manner. *Biomedicine & Pharmacotherapy*.

[46] Cavalcante M., Costa G., Arago G. F., Guimares S., Vasconcelos P. (2021). Nutraceutical immunomodulation on acute inflammation by abdominal plastic surgery. *Journal of Pharmaceutical Negative Results*.

[47] Faden A. I., Wu J., Stoica B. A., Loane D. J. (2016). Progressive inflammation-mediated neurodegeneration after traumatic brain or spinal cord injury. *British Journal of Pharmacology*.

[48] Colonna M., Butovsky O. (2017). Microglia function in the central nervous system during health and neurodegeneration. *Annual Review of Immunology*.

[49] Cheng Y., Song Y., Chen H. (2021). Ferroptosis mediated by lipid reactive oxygen species: a possible causal link of neuroinflammation to neurological disorders. *Oxidative Medicine and Cellular Longevity*.

[50] Martinez F. O., Gordon S., Locati M., Mantovani A. (2006). Transcriptional profiling of the human monocyte-to-macrophage differentiation and polarization: new molecules and patterns of gene expression. *Journal of Immunology*.

[51] Loane D. J., Kumar A. (2016). Microglia in the TBI brain: the good, the bad, and the dysregulated. *Experimental Neurology*.

[52] Lin C.-L., Dumont A. S., Zhang J. H., Zuccarello M., Chen C.-S. (2017). Improving and predicting outcomes of traumatic brain injury: Neuroplasticity, Imaging Modalities, and Perspective Therapy. *Neural Plasticity*.

[53] Delage C., Taib T., Mamma C., Lerouet D., Besson V. C. (2021). Traumatic brain injury: an age-dependent view of post-traumatic neuroinflammation and its treatment. *Pharmaceutics*.

[54] Barbiero J. K., Santiago R., Tonin F. S. (2014). PPAR-*α* agonist fenofibrate protects against the damaging effects of MPTP in a rat model of Parkinson's disease. *Progress in Neuro-Psychopharmacology & Biological Psychiatry*.

[55] Polak P. E., Kalinin S., Dello Russo C. (2005). Protective effects of a peroxisome proliferator-activated receptor-*β*/*δ* agonist in experimental autoimmune encephalomyelitis. *Journal of Neuroimmunology*.

[56] Shao Z. Q., Liu Z. J. (2015). Neuroinflammation and neuronal autophagic death were suppressed via rosiglitazone treatment: new evidence on neuroprotection in a rat model of global cerebral ischemia. *Journal of the Neurological Sciences*.

[57] Ricote M., Glass C. K. (2007). PPARs and molecular mechanisms of transrepression. *Biochimica et Biophysica Acta*.

[58] Shang J., Brust R., Mosure S. A. (2018). Cooperative cobinding of synthetic and natural ligands to the nuclear receptor PPAR*γ*. *eLife*.

[59] Liu Q., Shan P., Li H. (2019). Gambogic acid prevents angiotensin II-induced abdominal aortic aneurysm through inflammatory and oxidative stress dependent targeting the PI3K/Akt/mTOR and NF-*κ*B signaling pathways. *Molecular Medicine Reports*.

[60] Cheng S.-C., Huang W.-C., Pang J.-H. S., Wu Y.-H., Cheng C.-Y. (2019). Quercetin inhibits the production of IL-1*β*-induced inflammatory cytokines and chemokines in ARPE-19 cells via the MAPK and NF-*κ*B signaling pathways. *International Journal of Molecular Sciences*.

[61] Paige E., Clément M., Lareyre F. (2019). Interleukin-6 receptor signaling and abdominal aortic aneurysm growth rates. *Circulation: Genomic and Precision Medicine*.

[62] Sauerbeck A., Gao J., Readnower R. (2011). Pioglitazone attenuates mitochondrial dysfunction, cognitive impairment, cortical tissue loss, and inflammation following traumatic brain injury. *Experimental Neurology*.

[63] Thal S. C., Heinemann M., Luh C., Pieter D., Werner C., Engelhard K. (2011). Pioglitazone reduces secondary brain damage after experimental brain trauma by PPAR-*γ*-independent mechanisms. *Journal of Neurotrauma*.

[64] Poojary R., Kumar N. A., Kumarchandra R. (2020). Assessment of monoamine neurotransmitters in the cortex and cerebellum of gamma-irradiated mice: a neuromodulatory role of Cynodon dactylon. *Journal of Carcinogenesis*.

[65] Bernardo A., Minghetti L. (2008). Regulation of glial cell functions by PPAR-gamma natural and synthetic agonists. *PPAR Research*.

[66] Hosni A. A., Abdel-Moneim A. A., Abdel-Reheim E. S., Mohamed S. M., Helmy H. (2017). Cinnamaldehyde potentially attenuates gestational hyperglycemia in rats through modulation of PPAR*γ*, proinflammatory cytokines and oxidative stress. *Biomedicine & Pharmacotherapy*.

[67] Beheshti F., Hosseini M., Hashemzehi M., Soukhtanloo M., Khazaei M., Shafei M. N. (2019). The effects of PPAR-*γ* agonist pioglitazone on hippocampal cytokines, brain-derived neurotrophic factor, memory impairment, and oxidative stress status in lipopolysaccharide-treated rats. *Iranian Journal of Basic Medical Sciences*.

[68] Kaplan J., Nowell M., Chima R., Zingarelli B. (2014). Pioglitazone reduces inflammation through inhibition of NF-*κ*B in polymicrobial sepsis. *Innate Immunity*.

[69] Wei X., Hu C. C., Zhang Y. L., Yao S. L., Mao W. K. (2016). Telmisartan reduced cerebral edema by inhibiting NLRP3 inflammasome in mice with cold brain injury. *Journal of Huazhong University of Science and Technology. Medical Sciences*.

[70] Qian H., Li Q., Shi W. (2017). Hyperbaric oxygen alleviates the activation of NLRP-3-inflammasomes in traumatic brain injury. *Molecular Medicine Reports*.

[71] Saadi M., Karkhah A., Pourabdolhossein F., Ataie A., Monif M., Nouri H. R. (2020). Involvement of NLRC4 inflammasome through caspase-1 and IL-1*β* augments neuroinflammation and contributes to memory impairment in an experimental model of Alzheimer's like disease. *Brain Research Bulletin*.

[72] O'Brien W. T., Pham L., Symons G. F., Monif M., Shultz S. R., McDonald S. J. (2020). The NLRP3 inflammasome in traumatic brain injury: potential as a biomarker and therapeutic target. *Journal of Neuroinflammation*.

[73] Yi H. J., Lee J. E., Lee D. H. (2020). The role of NLRP3 in traumatic brain injury and its regulation by pioglitazone. *Journal of Neurosurgery*.

[74] Glatz T., Stöck I., Nguyen-Ngoc M. (2010). Peroxisome-proliferator-activated receptors gamma and peroxisome-proliferator-activated receptors beta/delta and the regulation of interleukin 1 receptor antagonist expression by pioglitazone in ischaemic brain. *Journal of Hypertension*.

[75] Qiu D., Li X. N. (2015). Pioglitazone inhibits the secretion of proinflammatory cytokines and chemokines in astrocytes stimulated with lipopolysaccharide. *International Journal of Clinical Pharmacology and Therapeutics*.

[76] Das M., Leonardo C. C., Rangooni S., Pennypacker K. R., Mohapatra S., Mohapatra S. S. (2011). Lateral fluid percussion injury of the brain induces CCL20 inflammatory chemokine expression in rats. *Journal of Neuroinflammation*.

[77] Leonardo C. C., Musso J., Das M. (2012). CCL20 is associated with neurodegeneration following experimental traumatic brain injury and promotes cellular toxicity in vitro. *Translational Stroke Research*.

[78] Kim H.-J., Lee J.-H., Kim S.-H. (2010). Therapeutic effects of human mesenchymal stem cells on traumatic brain injury in rats: secretion of neurotrophic factors and inhibition of apoptosis. *Journal of Neurotrauma*.

[79] Korley F. K., Diaz-Arrastia R., Wu A. H. (2016). Circulating brain-derived neurotrophic factor has diagnostic and prognostic value in traumatic brain injury. *Journal of Neurotrauma*.

[80] Robertson C. L., Soane L., Siegel Z. T., Fiskum G. (2006). The potential role of mitochondria in pediatric traumatic brain injury. *Developmental Neuroscience*.

[81] Hunter R. L., Dragicevic N., Seifert K. (2007). Inflammation induces mitochondrial dysfunction and dopaminergic neurodegeneration in the nigrostriatal system. *Journal of Neurochemistry*.

[82] McTigue D. M., Tripathi R., Wei P., Lash A. T. (2007). The PPAR gamma agonist pioglitazone improves anatomical and locomotor recovery after rodent spinal cord injury. *Experimental Neurology*.

[83] Qiu L., Jiang X., Wen L., Hu Q., Deng Y. (2016). Pioglitazone decreases the levels of inflammatory cytokines in SD rats with traumatic brain injury via up-regulating PPAR*γ*. *Xi Bao Yu Fen Zi Mian Yi Xue Za Zhi*.

[84] Yang H., Chen C. (2008). Cyclooxygenase-2 in synaptic signaling. *Current Pharmaceutical Design*.

[85] Bazmandegan G., Boroushaki M. T., Shamsizadeh A., Ayoobi F., Hakimizadeh E., Allahtavakoli M. (2017). Brown propolis attenuates cerebral ischemia-induced oxidative damage via affecting antioxidant enzyme system in mice. *Biomedicine & Pharmacotherapy*.

[86] Pilipović K., Župan Ž., Dolenec P., Mršić-Pelčić J., Župan G. (2015). A single dose of PPAR*γ* agonist pioglitazone reduces cortical oxidative damage and microglial reaction following lateral fluid percussion brain injury in rats. *Progress in Neuro-Psychopharmacology & Biological Psychiatry*.

